# Nitrogen Removal Characteristics and Application by a Novel Cold-Resistant Bacterium

**DOI:** 10.3390/microorganisms14071529

**Published:** 2026-07-13

**Authors:** Wanqiu Chen, Xiaoling Li, Xianling Nie, Jun Cheng, Hui Jin, Jifa Liu, Jianqiang Zhao, Xiaohong Zhao

**Affiliations:** 1 Chang’an University, Xi’an 710054, China; wanqiuc@chd.edu.cn (W.C.); chdnxl@163.com (X.N.); jinhui20020315@163.com (H.J.); liujf@chd.edu.cn (J.L.); jqzhao@chd.edu.cn (J.Z.); xzhao@chd.edu.cn (X.Z.); 2 Henan Institute of Technology, Xinxiang 453003, China; 3 Shaanxi Water Development Environment Group Co., Ltd., Xi’an 710018, China; 17782833539@163.com

**Keywords:** *Acinetobacter pragensis*, cold-resistant, nitrogen assimilation, bioaugmentation, wastewater treatment

## Abstract

Biological nitrogen removal is often inhibited under low-temperature conditions due to reduced microbial activity and impaired metabolic processes. A novel cold-resistant bacterium, *Acinetobacter pragensis* FX1, was isolated and exhibited efficient nitrogen removal capacity across a broad temperature range of 5–30 °C. Notably, strain FX1 remained metabolically active at 5 °C, with a specific growth rate of 0.04 h^−1^ and a generation time of 18.09 h, demonstrating excellent cold-resistant capability. Under ideal operating conditions (sodium citrate as carbon source, C/N ratio of 10, pH 7, and a shaking speed of 150 rpm) at 10 °C, removal efficiencies of NH_4_^+^-N, NO_3_^−^-N, and NO_2_^−^-N reached 99.39%, 81.30%, and 99.78%, respectively. Nitrogen balance analysis, combined with genome annotation, revealed that nitrogen assimilation was the predominant pathway, thereby avoiding the potential for greenhouse gas (N_2_O) generation. Genes associated with cold shock response and fatty acid biosynthesis supported the maintenance of membrane stability and the sustainability of metabolic activity under cold stress. Furthermore, bioaugmentation with strain FX1 successfully improved nutrient removal performance at 10 °C, driven by synergistic interactions with indigenous microbial communities. Overall, these results demonstrate the potential of strain FX1 as a promising candidate for enhancing biological nitrogen removal performance in cold-region wastewater treatment.

## 1. Introduction

Microorganisms are fundamental drivers of environmental sustainability, participating in key biological processes and interactions with other organisms to regulate biogeochemical cycles and shape ecosystems [1]. Compared with physicochemical approaches, which are often associated with high operational costs and risks of secondary pollution, microbial biotechnology offers a more sustainable and environmentally friendly alternative [2,3]. Conventional biological nitrogen removal primarily relies on the sequential coupling of autotrophic nitrification and heterotrophic denitrification. However, the inherently slow growth of nitrifiers and the requirement for separated redox zones prolong hydraulic retention times and increase operational complexity [4]. In recent years, heterotrophic nitrification–aerobic denitrification (HNAD) bacteria have attracted increasing attention; they are capable of performing both nitrification and denitrification simultaneously without alternating aerobic and anoxic conditions, such as *Zobellella* sp. An-6 [5], *Acinetobacter haemolyticus* RH19 [6], *Agrobacterium* sp. KG3 and *Stutzerimonas* sp. KA4 [7]. During this process, nitrous oxide (N_2_O) could be generated as an intermediate or by-product. Wastewater treatment has been identified as one of the major anthropogenic sources of N_2_O, with average emissions in the United States estimated at 11.6 MMT CO_2_-eq [8]. As a potent greenhouse gas, N_2_O contributed approximately 6.4% of the total warming associated with greenhouse gases from 1750 to 2022 [9].

Ammonium assimilation represents an alternative microbial nitrogen transformation pathway by incorporating inorganic nitrogen directly into cellular biomass for amino acid, protein, and nucleic acid synthesis, which avoids the generation of N_2_O [10]. In contrast with the nitrification–denitrification pathway, this process offers a paradigm shift from “nitrogen removal” to “nitrogen retention and resource recovery”, shortens the nitrogen removal process, and potentially achieves higher nitrogen removal efficiency [11,12]. The nitrogen-rich biomass generated can be harvested as a renewable product (e.g., single-cell protein), aligning well with the principles of a low-carbon circular economy. Yet, limited studies have reported efficient strains simultaneously performing nitrogen removal and heterotrophic assimilation at low temperatures.

Temperature is a critical environmental factor for microbial growth rate and nitrogen transformation processes, particularly in cold regions [13,14]. Low temperatures significantly suppress microbial activity, substantially diminishing the degradation efficiency of nitrogenous pollutants and substandard effluent quality, thereby leading to eutrophication and threatening ecosystem and human health [15]. Although several cold-resistant bacteria have been isolated, such as *Janthinobacterium* sp. J1-1 [16], *Rhizobium* sp. WS7 [15], and *Acinetobacter calcoaceticus* TY1 [17], most of them rely on nitrification-denitrification pathways and may still generate N_2_O, thereby limiting their application for near-zero greenhouse gas emissions. Consequently, it is meaningful to explore these microbial resources that can retain ammonium efficiently in biomass at low temperatures, combining high nitrogen removal efficiency with minimal environmental impact.

In response to these challenges, a novel cold-resistant bacterium, *Acinetobacter pragensis* FX1, was isolated from activated sludge and investigated as a potential microbial resource for low-temperature wastewater treatment. Under different environmental conditions and nitrogen sources, the nitrogen removal performance and the growth characteristics were examined. Particularly at 5 °C, the capacity to maintain metabolic activity was assessed. Moreover, nitrogen balance analysis, together with whole-genome sequencing, was adopted to clarify the nitrogen transformation pathways. The genetic determinants associated with cold resistance were analyzed. In addition, bioaugmentation experiments were performed to evaluate whether strain FX1 could enhance nutrient removal through interactions with indigenous microbial communities. Through linking microbial physiology, metabolic mechanisms, and community-level responses, this study seeks to provide a sustainable strategy for improving wastewater treatment performance operating under low-temperature conditions.

## 2. Materials and Methods

### 2.1. Medium

The Luria–Bertani medium (LB), Bromothymol blue medium (BTB), basal enriched medium (BM), heterotrophic nitrification medium (HNM), Aerobic denitrification medium (ADM), and simultaneous nitrification and denitrification medium (SNDM) were used in screening bacteria and testing the performance. Components of the media are listed in Table S1.

### 2.2. Sample, Isolation, and Identification

The samples were collected from aerobic pond activated sludge of five municipal wastewater treatment plants in northern Shaanxi, China (Text S1). After washing and centrifuging, the sludge samples were added to the LB medium and incubated at 10 °C with constant shaking at 150 rpm for 48 h. Subsequently, 5 mL of the enriched culture was transferred into 100 mL of fresh BM and incubated under the same conditions for an additional 72 h to screen denitrifying bacteria. 100 μL of the 10^−1^–10^−8^ gradient dilution was plated onto solid BTB and incubated at 10 °C until distinct blue colonies appeared. Separate colonies were repeatedly streaked (5–6 times) on solid BTB to obtain purified strains. Purified strains were inoculated into BM, and the optical density at 600 nm (OD_600_) and NO_3_^−^-N concentration were determined after 72 h of cultivation as described in Section 2.8. The strain exhibiting the highest NO_3_^−^-N removal efficiency was selected for subsequent identification and characterization. The morphological characteristics of strain FX1 were examined by scanning electron microscopy (SEM; Regulus 8100, Hitachi High-Tech Corporation, Tokyo, Japan) (Text S2). Strain FX1 was identified by 16S rDNA gene sequencing at Scientific Compass (Hangzhou, China) (Text S3). The acquired sequences were compared with the known microbial sequences using the online BLAST (http://blast.ncbi.nlm.nih.gov/Blast.cgi, accessed on 18 April 2026), and uploaded to GenBank. The phylogenetic tree was generated by MEGA 7.0 based on the 16S rDNA sequences by the neighbor-joining method with bootstrap values of 1000 replications.

### 2.3. Different Parameters on Nitrate Removal

In order to determine the impacts of various culture conditions on the growth and nitrogen removal performance of strain FX1 at low temperature, single-factor experiments were performed with NO_3_^−^-N as the sole nitrogen source with an initial NO_3_^−^-N concentration of 50 mg/L at 10 °C. The strain was precultured in LB at 10 °C and 150 rpm for 48 h. The cells were harvested by centrifugation, washed thrice with sterile 1 × PBS, and resuspended in sterile ADM-1 to prepare the bacterial pre-incubated inoculum. Subsequently, 5.0 mL of the bacterial inoculum was transferred into 100 mL of ADM-1 in 250 mL flasks and incubated at 10 °C for 72 h under the test conditions. Single-factor assays investigated: carbon source types (sodium acetate, sodium succinate, sodium citrate, sodium oxalate), C/N ratios (5, 10, 15, and 20), pH values (6, 7, 8, and 9), shaking speed (50, 100, 150, and 200 rpm), and temperature (5, 10, 20, and 30 °C). Samples were taken periodically to monitor OD_600_ and NO_3_^−^-N concentration as described in Section 2.8.

Based on the obtained growth curves of strain FX1 under the above different temperature conditions, the growth rate and generation time were calculated, according to the following formulas [18]:

(1) $$k=\frac{\left(\ln\left(N2/N1\right)\right)}{t2-t1}$$

(2) $$g=\frac{\ln\left(2\right)}{k}$$

where *k* is the specific rate (h^−1^), *N*_1_ and *N*_2_ are the initial and terminal OD_600_ values on the maximum slope of the growth curve between corresponding initial and terminal times *t*_1_ and *t*_2_, respectively. *g* is generation time (h).

### 2.4. Determination of Nitrogen Removal Performance

The nitrogen removal capacity of strain FX1 at 10 °C was investigated using different media, which comprised a single nitrogen source (HNM, ADM-1, ADM-2) and a combined nitrogen source (SNDM-1, SNDM-2). The strain FX1 was pre-incubated as described in Section 2.3. The 100 mL tested medium was inoculated with 5 mL of the pre-incubated inoculum and cultured at 10 °C and 150 rpm for 72 h. OD_600_ and nutrient concentrations of NH_4_^+^-N, NO_2_^−^-N, NO_3_^−^-N, NH_2_OH, and chemical oxygen demand (COD) were tested regularly as described in Section 2.8.

### 2.5. Nitrogen Balance

Nitrogen transformation pathways were quantified using the nitrogen mass balance method described by Yan et al. [10]. Strain FX1 was cultivated (1%, *v*/*v*) in HNM, ADM-1, and ADM-2 media at 10 °C for 72 h. Samples collected at 0 and 72 h were centrifuged and filtered before the determination of NH_4_^+^-N, NO_2_^−^-N, and NO_3_^−^-N, and soluble total nitrogen (soluble TN_1_). Biomass-associated nitrogen was quantified from cell lysates obtained by ultrasonication (JY98-IIIDN, Scientz Biotechnology Co., Ltd., Ningbo, China) at 120 W for 5 min with a pulse regime of 3 s on and 2 s off in an ice bath. Total nitrogen released after cell disruption was defined as TN. Following centrifugation and filtration of the lysate, the total soluble nitrogen measured in the resulting supernatant was designated as TN_2_. Intracellular TN consists of Intracellular soluble TN and Biology nitrogen (intracellular solid TN), which were calculated according to the following equations. The analytical methods used for the determination of all nitrogen species are provided in Section 2.8.

(3) Nitrogen loss = Initial TN − Final TN

(4) Extracellular organic-N = Final soluble TN_1_ − Final (NH_4_^+^-N) − Final (NO_2_^−^-N) − Final (NO_3_^−^-N)

(5) Intracellular soluble TN = Final soluble TN_2_ − Final soluble TN_1_

(6) Biology nitrogen = Final TN − Final soluble TN_1_ − Intracellular soluble TN

where Initial TN and Final TN represent the total nitrogen measured after cell disruption at 0 h and 72 h, respectively; Final soluble TN_1_ represents the soluble total nitrogen measured in the supernatant of the bacterial suspension at 72 h (before cell disruption); Final soluble TN_2_ is the total soluble nitrogen in the supernatant obtained after centrifugation and filtration of the cell lysate at 72 h.

### 2.6. Complete Genome Sequencing

Samples of strain FX1 were collected at the mid-logarithmic growth phase. Genome sequencing was performed by Majorbio Bio-pharm Technology Co., Ltd. (Shanghai, China) using a combination of PacBio Revio and Illumina NovaSeq X PLUS (Text S4). Gene annotation and functional prediction were employed on multiple databases (including NR, GO, KEGG, COG, TCDB, CAZY, Pfam, and Swiss-Prot) and sequence alignment tools such as Blast2Go, Diamond, and HMMER3.

### 2.7. Bioaugmentation Test

The activated sludge was collected from a laboratory-scale sequencing batch reactor (SBR) that had been continuously operated at 10 °C with stable treatment performance. The samples were centrifuged at 5000 rpm for 5 min and washed three times with sterile 0.9% saline solution. Batch experiments were conducted in four flasks with a working volume of 500 mL. The initial NH_4_^+^-N concentration was adjusted to 100 mg/L using (NH_4_)_2_SO_4_, while sodium acetate was supplied as the external carbon source to obtain a C/N ratio of 10. The remaining composition followed that of the HNM. All reactors were operated at 10 °C and 150 rpm for 72 h. The control reactor (R0) contained activated sludge only (the mixed liquor suspended solids (MLSS) ≈ 3500 mg/L), whereas the bioaugmented reactors (R1, R2, and R3) were bioaugmented with strain FX1 at volumetric ratios of 10%, 25%, and 50%, respectively, relative to the sludge suspension. Before inoculation, strain FX1 was cultivated in LB medium to the logarithmic growth phase (OD_600_ ≈ 1.0). Samples were collected periodically to determine NH_4_^+^-N, COD, TN, and total phosphorus (TP) concentrations as described in Section 2.8. At 0 h and 72 h, sludge samples were collected for microbial community analysis. All microbial sequencing was conducted by Majorbio Bio-pharm Technology Co., Ltd. (Shanghai, China) (Text S5).

### 2.8. Analytical Methods

The concentrations of NH_4_^+^-N (Nano reagent spectrophotometry), NO_2_^−^-N (N-1-naphthalenyl-ethylenediamine photometry), NO_3_^−^-N (UV photometry), and TN (alkaline potassium persulfate method) were determined following standard methods [19]. NH_2_OH was tested according to the 8-quinolinol spectrophotometric method [20]. TP was measured by the ammonium molybdate spectrophotometry following potassium persulfate digestion. COD was quantified using a COD analysis kit (5B-3C V8, Beijing Lianhua Technology Co., Ltd., Beijing, China). Biomass growth was measured by measuring OD_600_ using a spectrophotometer (UV-1200, Macy Instruments Co., Ltd., Shanghai, China). The pH was monitored using a multi-parameter water quality analyzer (Multi 3630 IDS, WTW, Weilheim, Germany). All measurements were conducted in triplicate. Experimental data were presented as the mean ± standard deviation (SD), with error bars in the figures representing the SD. Statistical comparisons among bioaugmentation experiments were conducted using one-way analysis of variance (ANOVA) followed by Tukey’s test in SPSS 26.0, with significance levels of *p* < 0.05 (*), *p* < 0.01 (**), and *p* < 0.001 (***). Data processing and figure plotting were performed with Origin v.2024.

## 3. Results and Discussion

### 3.1. Isolation and Identification of Strain FX1

**Figure 1:**
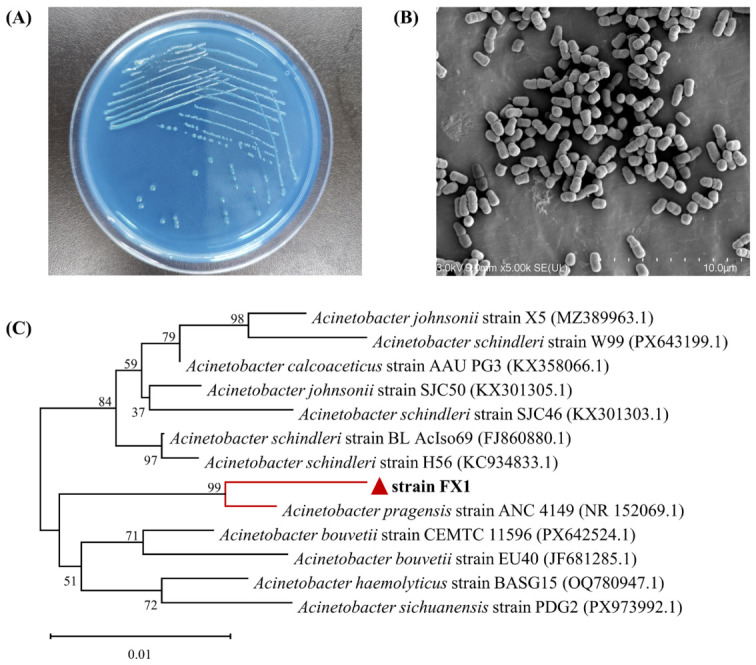
Identification results of strain FX1. Bacterial colonies (**A**); SEM (**B**); Phylogenetic tree (**C**).

A total of nine cold-resistant bacterial strains capable of nitrate removal were isolated (Figure S1). Among them, the strain FX1 exhibited the highest nitrogen removal capability and growth characteristics at 10 °C, achieving the highest NO_3_^−^-N removal efficiency of 82.94% and OD_600_ of 0.90. Therefore, strain FX1 was selected for subsequent characterization and application studies. The strain has been deposited in the China General Microbiological Culture Collection Center (CGMCC) under accession number CGMCC No. 36583. On BTB agar plates, strain FX1 formed round, white, and moist colonies with smooth surfaces, approximately 2–3 mm in diameter (Figure 1A). The scanning electron microscope revealed that the cells were short, rod-shaped (Figure 1B). The 16S rDNA gene of strain FX1 was sequenced and submitted to GenBank with the accession number PP976548. Phylogenetic analysis showed that strain FX1 clustered closely with *Acinetobacter pragensis* strain ANC 4149 (accession number: NR 152069.1), as illustrated in Figure 1C. Thus, strain FX1 was identified as a member of *Acinetobacter pragensis*.

### 3.2. Different Culturing Conditions for Nitrate Removal

**Figure 2:**
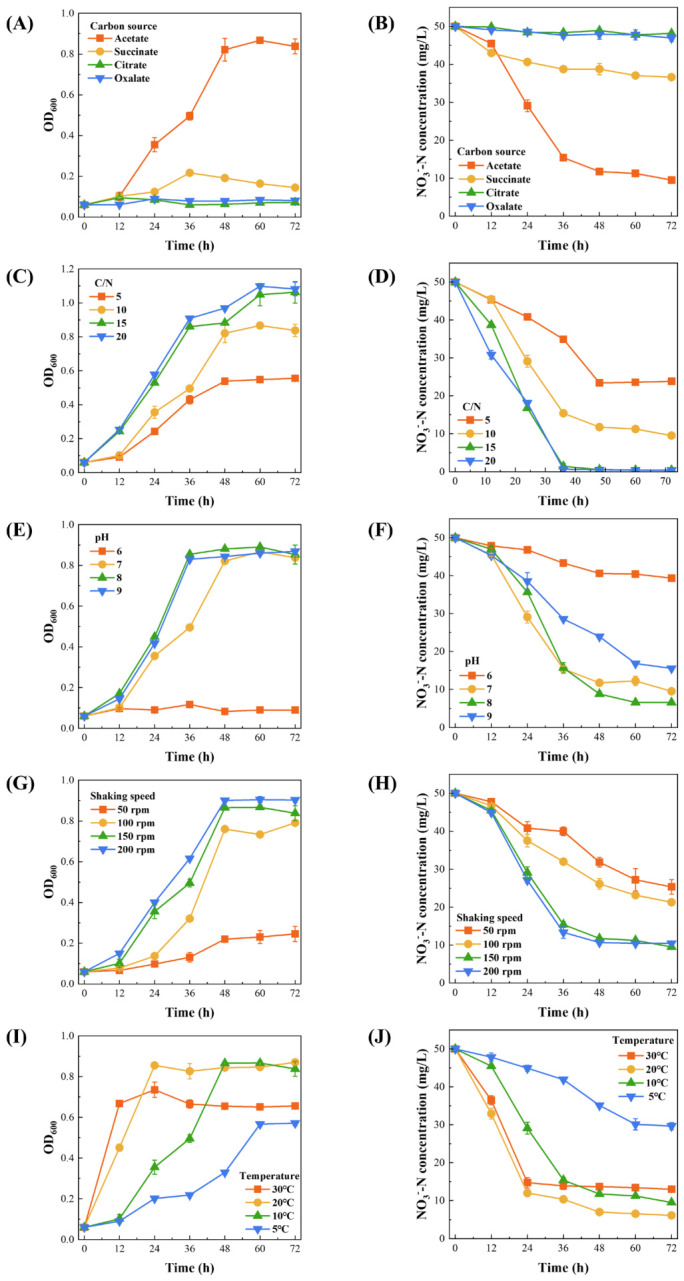
The growth and NO_3_^−^-N concentrations of strain FX1 incubated at different environmental conditions. Carbon source (**A**,**B**), C/N ratio (**C**,**D**), pH (**E**,**F**), shaking speed (**G**,**H**), and temperature (**I**,**J**).

Carbon source strongly influences microbial metabolism and nitrogen conversion performance [21]. As depicted in Figure 2A,B, strain FX1 exhibited distinct substrate preferences, with sodium acetate supporting both the highest NO_3_^−^-N removal efficiency and biomass accumulation. Following a lag phase of approximately 12 h, NO_3_^−^-N concentration decreased rapidly and reached 9.53 mg/L after 72 h, accompanied by an increase in OD_600_ to 0.84. Similar substrate preference has been reported for other bacteria, such as *Klebsiella* sp. TSH15 [22]. In contrast, NO_3_^−^-N removal and cell growth were substantially reduced when sodium succinate was supplied, resulting in a residual NO_3_^−^-N concentration of 36.62 mg/L and a maximum OD_600_ of only 0.22. Even lower growth and NO_3_^−^-N removal capacities were observed with sodium citrate and sodium oxalate. These preferences may be attributed to differences in the reducibility of distinct carbon source types and the genome repertoire of strain FX1 [23,24].

The effect of C/N ratio on NO_3_^−^-N removal and cell growth was further evaluated using sodium acetate as the sole carbon source (Figure 2C,D). At a C/N ratio of 5, NO_3_^−^-N removal efficiency reached only 53.20%, accompanied by a low OD_600_ of 0.54, indicating that carbon limitation restricted both bacterial growth and nitrogen assimilation. Increasing the C/N ratio to 10, NO_3_^−^-N removal was substantially enhanced to 80.94%, which suggests that a greater carbon supply provided sufficient energy to support biomass production and metabolism [25]. Further increases in C/N ratio to 15 and 20 resulted in nearly complete NO_3_^−^-N removal (99.13% and 99.22%, respectively), while biomass growth showed only slight additional improvement. That suggested the C/N ratio of 15 could be optimal for strain FX1. Nevertheless, to balance treatment performance and cost-effectiveness, a ratio of 10 was adopted in subsequent experiments.

As depicted in Figure 2E,F, both cell growth and nitrate removal by strain FX1 were strongly affected by pH. Under weakly acidic conditions at pH 6, the strain exhibited limited biomass accumulation (OD_600_ = 0.09) and poor NO_3_^−^-N removal performance, with a maximum removal efficiency of only 21.27% after 72 h. By contrast, strain FX1 performed well under neutral and mildly alkaline conditions. Similar growth patterns were observed at pH 7 and 8, characterized by a lag phase of approximately 12 h followed by rapid growth between 12 and 36 h. NO_3_^−^-N removal efficiency reached 80.94% and 86.82% at pH 7 and 8, respectively. The slightly enhanced performance at pH 8 may be associated with improved enzymatic activity and metabolic efficiency under mildly alkaline conditions, which facilitated cellular growth and NO_3_^−^-N removal. However, further increasing the pH to 9 resulted in a decline in both biomass accumulation and NO_3_^−^-N removal, indicating that excessive alkalinity imposed physiological stress on the strain and reduced the metabolic activity. These results indicated that neutral to mildly alkaline conditions (pH from 7 to 8) were the optimal range for strain FX1, which is similar to other reported strains, such as *Halomonas venusta* SND-01 [11] and *Bacillus* strain N31 [26]. Considering operational stability and practical wastewater treatment applications, the subsequent experiments were conducted at pH 7.

Dissolved oxygen availability was regulated by varying the shaking speed, and the influence on NO_3_^−^-N removal and cell growth was presented in Figure 2G,H. As the shaking speed increased from 50 rpm to 150 rpm, both NO_3_^−^-N removal efficiency and microbial growth steadily improved. The trend suggests that enhanced oxygen transfer and substrate availability under higher shaking speeds promoted cellular growth and metabolic activity [27,28], thereby facilitating NO_3_^−^-N utilization. At 150 rpm, strain FX1 achieved the highest NO_3_^−^-N removal efficiency (80.94%) with an OD_600_ of 0.87. When the shaking speed was further increased to 200 rpm, the NO_3_^−^-N removal efficiency and biomass accumulation remained at a similarly high level, reaching 79.11% and an OD_600_ of 0.90 at 72 h, respectively. The comparable performance observed between 150 and 200 rpm indicates that oxygen transfer was no longer a major limiting factor within this range. Previous studies have reported that increasing shaking speeds can enhance microbial activity only up to a certain threshold, beyond which further increases provide limited benefits and may even lead to reduced nitrogen transformation efficiency [28,29]. Considering both nitrogen removal performance and energy consumption, 150 rpm was selected as the optimal shaking speed for subsequent experiments.

Temperature is widely considered as a major environmental stress factor for biological systems. Either at excessively high or low temperatures, which could disrupt metabolic balance and constrain functional performance, resulting in pronounced variability in pollutant removal efficiency among microbial strains [30]. As presented in Figure 2I,J, strain FX1 could thrive and maintain nitrogen removal activity over a broad temperature range of 5–30 °C. Strain FX1 exhibited stronger low-temperature tolerance compared to the psychrophilic strain *Pseudomonas weihenstephanensis* YB1107, which remained active between 8 and 30 °C but completely lost growth and nitrogen removal capacity at 6 °C [31]. Such broad temperature adaptability highlights the potential of strain FX1 for wastewater treatment systems subjected to seasonal temperature fluctuations. Notably, strain FX1 achieved 40.65% NO_3_^−^-N removal at 5 °C, substantially higher than the 27.22% reported for the psychrophilic bacterium *Bacillus simplex* H-b [32], highlighting the strong capacity for nitrogen removal under cold conditions. When the temperature was increased to 10 °C, both biomass accumulation and NO_3_^−^-N removal improved significantly, with OD_600_ and removal efficiency of 0.87 and 80.94%, respectively. The removal efficiency at 10 °C was nearly twice that observed at 5 °C. As the temperature further rose to 20 °C, the strain grew rapidly, with a substantially shortened lag phase and the highest removal efficiency of 87.76%. However, when the temperature was increased to 30 °C, OD_600_ decreased to 0.66, accompanied by a reduction in NO_3_^−^-N removal efficiency to 73.99%. This result indicates that temperatures above the physiological optimum imposed metabolic stress that constrained both growth and nitrogen conversion. That similar trends have been reported for other cold-resistant bacteria, strain FX1 retained substantial metabolic activity at low temperatures, whereas the maximum growth was observed at 20 °C [33,34].

As listed in Table 1, the specific growth rate of strain FX1 rose progressively from 0.04 h^−1^ at 5 °C to 0.20 h^−1^ at 30 °C, and the generation time decreased from 18.09 h to 3.46 h, indicating that elevated temperatures accelerated cell division and population turnover. Despite exhibiting the shortest generation time (3.46 h) and highest specific growth rate (0.20 h^−1^) at 30 °C, strain FX1 achieved a lower biomass concentration (maximum OD_600_ = 0.74) than that observed at 20 °C (maximum OD_600_ = 0.87) and 10 °C (maximum OD_600_ = 0.87). This discrepancy suggests a temperature-dependent trade-off relationship in cellular resource allocation. At elevated temperatures, a larger proportion of metabolic energy may be diverted from biomass synthesis toward cellular maintenance and stress adaptation processes [35], thereby reducing biomass yield. In contrast, moderate temperatures (10–20 °C) appeared to provide a more favorable balance between cell proliferation and biomass production of strain FX1, ultimately supporting higher biomass accumulation and NO_3_^−^-N removal performance.

### 3.3. Nitrogen Removal Performance of Strain FX1

#### 3.3.1. The Sole Nitrogen Source

**Figure 3:**
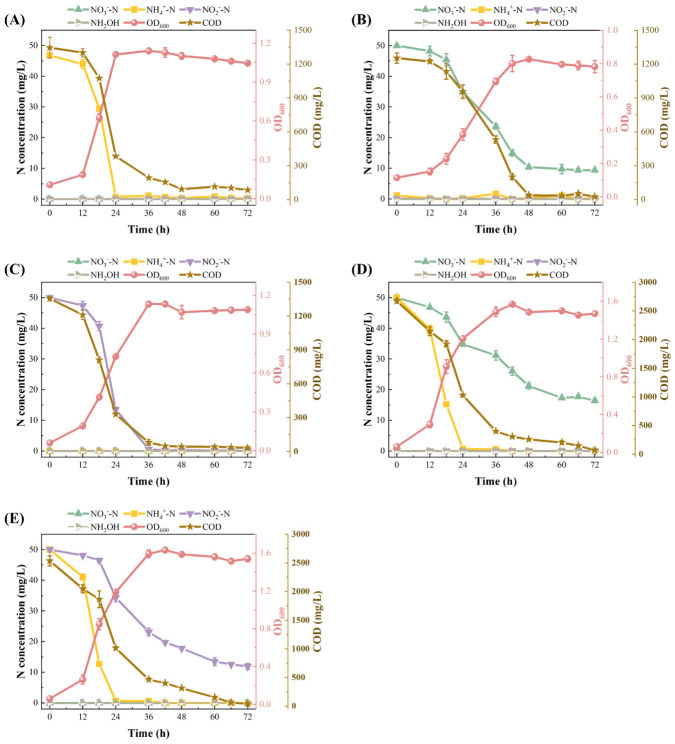
Growth characteristics and purification performance of strain FX1 when NH_4_^+^-N (**A**), NO_3_^−^-N (**B**), NO_2_^−^-N (**C**), NH_4_^+^-N + NO_3_^−^-N (**D**), and NH_4_^+^-N + NO_2_^−^-N (**E**) were used as the nitrogen source.

HNM was employed to evaluate the NH_4_^+^-N removal performance of strain FX1 at 10 °C (Figure 3A). During an initial 12 h lag phase, the strain exhibited slow growth, with OD_600_ increasing from 0.11 to 0.19. Following this adaptation period, NH_4_^+^-N was rapidly removed between 12 and 24 h, achieving an average removal rate of 3.61 mg/L/h, accompanied by a sharp increase in biomass (OD_600_ = 1.11). This removal rate was superior to that reported for *Glutamicibacter halophytocola* MD1 (1.82 mg/L/h) [36] at 30 °C, demonstrating the remarkably low-temperature nitrogen removal capacity of strain FX1. Notably, no detectable accumulation of NH_2_OH, NO_2_^−^-N, or NO_3_^−^-N was observed throughout the cultivation period, indicating that NH_4_^+^-N removal might be predominantly driven by assimilatory metabolism rather than conventional nitrification. This observation is consistent with the assimilation-dominated mechanism reported for *Rhodococcus erythropolis* Y10, where NH_4_^+^-N is predominantly incorporated into biomass without intermediate product formation [37]. Ultimately, the removal efficiencies for NH_4_^+^-N and COD reached 99.39% and 93.69%, respectively, highlighting the potential of strain FX1 for simultaneous carbon and NH_4_^+^-N removal under low-temperature conditions.

When NO_3_^−^-N was supplied as the sole nitrogen source, strain FX1 remained capable of efficient growth and NO_3_^−^-N utilization under aerobic conditions with the highest removal efficiency of 81.30% (Figure 3B). Compared to the medium with NH_4_^+^-N, a slightly longer lag phase (approximately 18 h) was observed, indicating that additional metabolic adaptation was required for distinct inorganic nitrogen forms. In the subsequent period, NO_3_^−^-N concentration rapidly decreased from 45.39 mg/L at 18 h to 10.38 mg/L at 48 h, corresponding to an average removal rate of 1.17 mg/L/h. This removal rate was higher than that reported for *Rhodococcus* sp. CPZ24 (0.93 mg/L/h) at 30 °C [38], indicating the strong nitrate-reducing capability of strain FX1 under low-temperature conditions. Although higher NO_3_^−^-N removal rates have been reported for *Pseudomonas* sp. G16 (2.87 mg/L/h at 15 °C) [39], it suffered from a relatively long adaptation period (approximately 48 h) and was susceptible to nitrite inhibition. As previously reported, NO_2_^−^-N is generally regarded as a critical intermediate in biological nitrogen transformation and may exert toxic effects on microbial activity [40]. By contrast, when NO_2_^−^-N was applied as the sole nitrogen source, strain FX1 showed a shorter adaptation period (12 h) and maintained stable nitrogen removal activity (Figure 3C). During the logarithmic growth phase (12–36 h), NO_2_^−^-N was removed at an average rate of 1.95 mg/L/h, substantially higher than that of *Bacillus* sp. N31 (0.73 mg/L/h) [26] at 30 °C. By the end of cultivation, the highest NO_2_^−^-N removal efficiency reached 99.78%. The coupling between NO_2_^−^-N removal and biomass formation exhibited the strong tolerance of the strain to nitrite stress, which may be related to the metabolic pathway, specific enzyme systems, and gene regulation.

#### 3.3.2. Mixed Nitrogen Source

When NH_4_^+^-N and NO_3_^−^-N were simultaneously supplied, strain FX1 exhibited enhanced growth at the maximum OD_600_ reaching 1.57 (Figure 3D), indicating that the coexistence of NH_4_^+^-N and NO_3_^−^-N was conducive to promoting bacterial growth. Similar growth-promoting effects of mixed nitrogen substrates have been reported for *Pseudomonas fragi* EH-H1 [41]. NH_4_^+^-N was completely removed within 24 h, while NO_3_^−^-N was removed concurrently but at a markedly slower rate, with only 30.36% eliminated during the first 24 h. Unlike *Acinetobacter junii* ZHG-1, which initiates NO_3_^−^-N utilization only after NH_4_^+^-N depletion [42], strain FX1 could utilize these nitrogen species simultaneously. In addition, a clear preference for NH_4_^+^-N was evident. Following NH_4_^+^-N exhaustion, NO_3_^−^-N removal continued and ultimately reached 67.21% at 72 h, suggesting that strain FX1 possesses metabolic flexibility in response to changes in nitrogen availability.

A similar growth-promoting effect was observed when NH_4_^+^-N and NO_2_^−^-N were provided as mixed nitrogen sources (Figure 3E). The maximum biomass concentration (OD_600_ = 1.63) was achieved, slightly exceeding that obtained in the NH_4_^+^-N and NO_3_^−^-N system. NH_4_^+^-N removal was largely unaffected by the presence of NO_2_^−^-N, with 98.67% removed within 24 h, closely matching the performance observed with NH_4_^+^-N as the sole nitrogen source. This behavior contrasts with that reported for *Comamonas testosteroni* HR5, in which NO_2_^−^-N toxicity significantly inhibited NH_4_^+^-N removal under mixed nitrogen conditions [43]. Moreover, NO_2_^−^-N was gradually consumed, with the most rapid removal occurring between 18 and 60 h, resulting in a final removal efficiency of 76.18%.

To summarize, strain FX1 could efficiently utilize both sole and mixed nitrogen sources for bacterial growth, while exhibiting a pronounced preference for NH_4_^+^-N over NO_3_^−^-N and NO_2_^−^-N. This substrate preference is likely related to metabolic regulation and energetic constraints. Specifically, NH_4_^+^-N can be directly incorporated into cellular biomass via assimilation pathways, whereas NO_3_^−^-N and NO_2_^−^-N must first undergo reduction to NH_4_^+^-N prior to assimilation, which incurs additional energy demand. Therefore, NH_4_^+^-N serves as the dominant nitrogen source for biosynthesis when available. Collectively, these results highlight the applicability of strain FX1 in low-temperature wastewater treatment systems characterized by the coexistence of multiple nitrogen species.

### 3.4. Nitrogen Balance Analysis

A nitrogen balance analysis was carried out on strain FX1 to clarify nitrogen transformation pathways, as illustrated in Table 2. When NH_4_^+^-N was supplied as the sole nitrogen source, 24.21 ± 1.29 mg/L of the nitrogen was converted into intracellular soluble nitrogen, while 19.33 ± 0.14 mg/L was assimilated into biomass nitrogen. Based on the nitrogen balance analysis, negligible nitrogen loss was observed, indicating that assimilatory ammonium uptake was the dominant pathway, simultaneously supporting cellular growth and intracellular nitrogen storage. Similarly, when NO_3_^−^-N or NO_2_^−^-N served as the sole nitrogen source, no significant measurable nitrogen loss was detected, suggesting that assimilatory nitrate reduction was the primary transformation pathway, whereby NO_3_^−^-N and NO_2_^−^-N were first reduced to NH_4_^+^-N and subsequently assimilated into biomass. In addition, since no significant nitrogen loss was observed, gaseous products were not further analyzed for composition. Biology nitrogen formation was 14.55 ± 2.06 mg/L for NO_3_^−^-N and 15.31 ± 2.09 mg/L for NO_2_^−^-N, both markedly lower than that obtained with NH_4_^+^-N as the substrate (19.33 ± 0.14 mg/L), further indicating that ammonium was the preferred substrate for biomass synthesis. Across all nitrogen conditions tested, a larger fraction of assimilated nitrogen was allocated to intracellular soluble nitrogen pools rather than structural biomass. This suggests that a conserved physiological strategy in strain FX1 may facilitate intracellular nitrogen storage and enhance metabolic flexibility under fluctuating nitrogen availability. This suggests that a conserved physiological strategy in strain FX1, in which assimilated nitrogen may be preferentially stored in intracellular reservoirs rather than immediately committed to cell construction. Such a strategy could enhance cellular resilience under fluctuating nitrogen availability, enabling metabolic activation. Overall, strain FX1 can remove nitrogen via assimilatory ammonium uptake and assimilatory nitrate reduction. This metabolic profile avoids nitrogen loss and greenhouse gas emissions, thereby representing a sustainable and environmentally friendly strategy for concurrent wastewater treatment and nitrogen recovery.

### 3.5. Genome Sequencing and Analysis of Strain FX1

#### 3.5.1. Genomic Analysis

**Figure 4:**
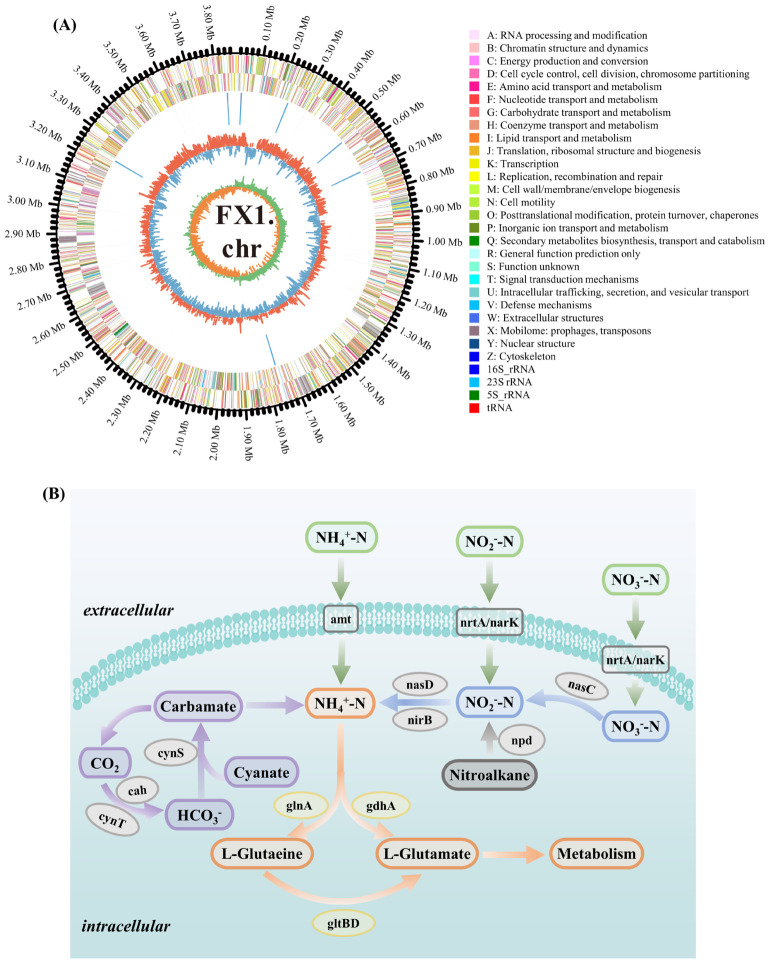
The circular genome maps of strain FX1 chromosome (**A**), with annotations from the outermost to the innermost circles: the genome size scale (outermost circle), CDSs on the positive and negative strands (second and third circles), rRNA and tRNA (fourth circle), GC content (fifth circle), and GC-skew values (innermost circle); Nitrogen metabolism pathways (**B**).

Whole-genome sequencing was performed to investigate the nitrogen metabolic potential and cold-resistant traits of strain FX1. The assembled genome has been deposited in the NCBI database under BioProject accession number PRJNA1395770. Genomic characterization revealed that strain FX1 possesses a genome size of 4,054,481 bp with a GC content of 43.68% (Table 3). The complete genome of strain FX1 comprises one circular chromosome (Figure 4A) and two circular plasmids (Figure S2), containing 21 rRNAs and 86 tRNA genes. The total length of the coding region was 3,465,834 bp, comprising 3884 predicted coding sequences (CDS), which accounted for 85.48% of the genome length.

#### 3.5.2. Analysis of Functional Genes for Nitrogen Metabolism

Functional annotation of the predicted CDSs was performed using the NR, Swiss-Prot, Pfam, COG, GO, and KEGG databases, resulting in 3860, 2635, 3131, 2892, 2658, and 2591 annotated genes, respectively. Functional annotation of predicted CDSs revealed genes associated with multiple nitrogen metabolic pathways, including assimilatory nitrate reduction (ANRA), dissimilatory nitrate reduction to ammonium (DNRA), ammonium assimilation, and auxiliary nitrogen utilization routes (Table S2). The corresponding nitrogen metabolic network is presented in Figure 4B.

Ammonium assimilation plays an essential role in coordinating carbon and nitrogen metabolism within bacterial cells [43]. NH_4_^+^-N uptake is mediated by ammonium transporters (amt), followed by assimilation into L-glutamate via the glutamine synthetase/glutamate synthase (*glnA* and *gltBD*) pathway or glutamate dehydrogenase (*gdhA*). L-glutamate functions as a central nitrogen donor for the biosynthesis of cellular nitrogenous compounds [44]. For oxidized nitrogen species, nitrate/nitrite transporters (*nrtA* and *narK*) facilitate substrate uptake. In the ANRA pathway, NO_3_^−^-N is reduced to NO_2_^−^-N by assimilatory nitrate reductase (*nasC*), followed by conversion to NH_4_^+^-N via nitrite reductase (*nasD*). However, the ecological contribution of ANRA is generally neglected, as microbial systems preferentially utilize NH_4_^+^-N for biomass synthesis [45]. In the DNRA pathway, NO_2_^−^-N is reduced to NH_4_^+^-N via nitrite reductase (*nirB*), after which ammonium is assimilated into biomass [46]. The presence of key genes involved in ammonium assimilation further supports the experimentally observed nitrogen conversion pattern. Importantly, genes typically associated with nitrification and denitrification were not detected in the genome of strain FX1. Consistently, experimental results showed no accumulation of NH_2_OH, NO_2_^−^-N, or NO_3_^−^-N during NH_4_^+^-N removal (see Section 3.3.1), and nitrogen balance analysis confirmed negligible gaseous nitrogen production under all conditions. The strong agreement between genomic features and physiological performance indicates that inorganic nitrogen is predominantly retained through assimilation rather than lost via gaseous pathways. This metabolic strategy enables efficient nitrogen recovery in the form of microbial biomass while minimizing nitrogen loss and avoiding greenhouse gas emissions, highlighting the environmental advantages of strain FX1 in wastewater treatment.

In addition, auxiliary nitrogen-related metabolic genes, including cyanate lyase (*cynS*), carbonic anhydrase (*cynT/cah*), and nitronate monooxygenase (*npd*), were identified. Cyanate degradation via *cynS* and *cynT/cah* provides an additional ammonium source for cellular assimilation [47], whereas *npd*-mediated nitroalkane oxidation may release NO_2_^−^-N, further expanding the spectrum of utilizable nitrogen substrates. Collectively, these auxiliary pathways suggest metabolic versatility in accessing diverse nitrogen forms, which may enhance nitrogen retention efficiency and biomass productivity in engineered systems.

#### 3.5.3. Analysis of Functional Genes for Cold-Resistant Metabolism

The strong low-temperature performance of strain FX1 observed in batch experiments (see Section 3.3), where efficient nitrogen removal was maintained even at 5 °C, suggests the presence of adaptive mechanisms that support cellular activity under cold stress. Genomic analysis further identified several genes potentially related to cold resistance, primarily those involved in cold shock response and the regulation of membrane fatty acid composition (Table S3). Among these, the cold shock protein gene *cspA* was identified in the genome of strain FX1. Previous studies have demonstrated that *cspA* contributes to the preservation of cellular homeostasis and metabolic function under low-temperature conditions through the regulation of stress-responsive pathways and protein synthesis [48,49]. Thus, the presence of *cspA* may help sustain these nitrogen-conversion processes by alleviating the inhibitory effects of low temperature on gene expression and protein synthesis. Genes involved in fatty acid biosynthesis were also identified, suggesting membrane properties could be regulated in response to cold stress [50,51]. Low temperatures generally reduce membrane fluidity, thereby impairing substrate uptake and intracellular metabolic processes. To counteract these effects, bacteria could remodel membrane lipids through modification of fatty acid chain lengths and membrane flexibility to preserve membrane functionality and maintain appropriate fluidity under cold conditions [52]. This coordinated activation of cold shock response genes and membrane adaptation-related pathways likely supports the maintenance of nitrogen metabolism at low temperatures, providing a potential mechanistic basis for the promising performance of strain FX1 in low-temperature wastewater treatment applications.

### 3.6. Application of Bioaugmentation with Strain FX1

**Figure 5:**
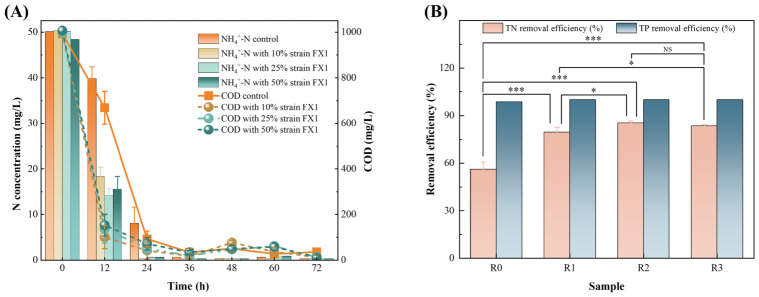
Pollutant removal performance of the activated sludge bioaugmented with different inoculation ratios of strain FX1: NH_4_^+^-N and COD (**A**), TN and TP (**B**). Statistical significance is indicated as *p* < 0.05 (*), *p* < 0.01 (**) and *p* < 0.001 (***), while NS indicates no significant.

To assess the feasibility of strain FX1 for low-temperature bioaugmentation, activated sludge systems were inoculated with strain FX1 at volumetric ratios of 10%, 25%, and 50% and operated at 10 °C. The seed sludge originated from a laboratory-scale SBR continuously operated under low temperature, ensuring stable nitrogen removal performance before the experiments (see Section 2.7). As shown in Figure 5A, the addition of strain FX1 markedly improved NH_4_^+^-N removal compared with the control group (R0). During the first 12 h, NH_4_^+^-N removal in R0 reached only 20.44%, reflecting the substantial inhibition of indigenous microbial activity under low-temperature conditions. In contrast, NH_4_^+^-N removal efficiencies increased to 63.73%, 71.81%, and 67.99% in R1, R2, and R3, respectively, indicating the rapid establishment of nitrogen transformation capacity following strain FX1 inoculation. Complete NH_4_^+^-N removal was achieved within 24 h in all bioaugmented systems, whereas approximately 36 h was required in R0. These findings demonstrate that even the low inoculation ratio was sufficient to substantially accelerate ammonium removal under cold conditions, highlighting the strong low-temperature adaptability and functional competitiveness of strain FX1 in activated sludge. The removal trend of COD was consistent with NH_4_^+^-N degradation, suggesting that carbon utilization and nitrogen transformation were concurrently stimulated after strain FX1 introduction. Compared with R0 (56.14%), all bioaugmented reactors exhibited significantly higher TN removal efficiencies (*p* < 0.001), reaching 79.58%, 85.41%, and 83.42% in R1, R2, and R3, respectively (Figure 5B). At lower inoculation levels, increasing FX1 input from10% to 25% could enhance the functional contribution to TN removal (*p* < 0.05). However, further increases beyond 25% did not lead to additional improvement. These results indicate that moderate inoculation (25%) may be sufficient to achieve near-maximum TN removal efficiency. Meanwhile, near-complete TP removal was ultimately achieved in all reactors, implying that phosphorus removal was less affected by low-temperature stress than nitrogen removal in the present systems. The improved nitrogen removal performance following strain FX1 inoculation was likely attributable to its strong cold resistance and efficient assimilation metabolism. In contrast to conventional nitrification-denitrification processes, which are often constrained by reduced enzymatic activity at low temperatures, assimilation of nitrogen is generally associated with lower metabolic requirements and can therefore remain active under cold stress. This metabolic characteristic may enable strain FX1 to sustain efficient nitrogen assimilation and partially compensate for the reduced activity of indigenous microorganisms, thereby accelerating nutrient removal.

**Figure 6:**
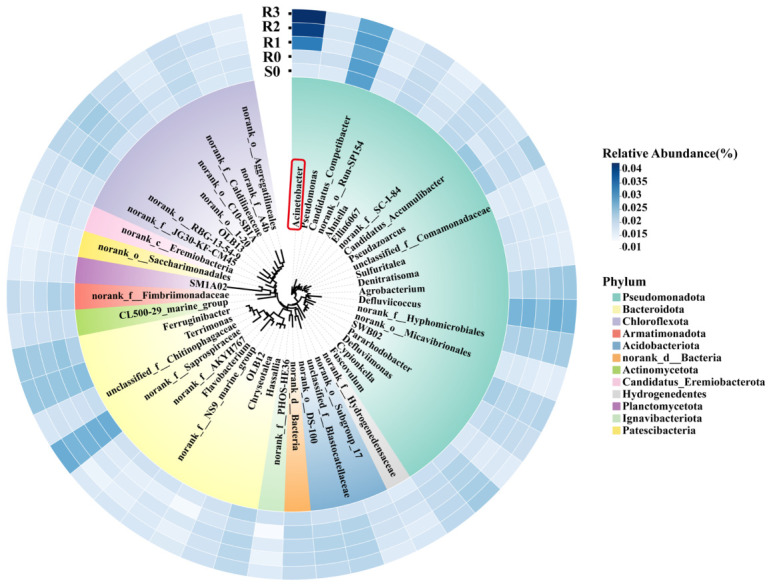
Microbial composition and relative abundance: R0, R1, R2, and R3 at 72 h; S0 at 0 h.

Consistent with the enhanced nutrient removal under low-temperature conditions, microbial community analysis revealed distinct shifts in community composition following strain FX1 inoculation. At the phylum level, Pseudomonadota remained dominant across all samples, with a markedly higher relative abundance in the bioaugmented groups than in R0 (Figure 6). The microbial community structure of R0 closely resembled that of the inoculated sludge (S0), further indicating that the sludge community had already developed a stable state following long-term acclimation to 10 °C. More notable community succession was observed at the genus level. The relative abundance of *Acinetobacter* increased from 0.47% to 0.79% in R0. In contrast, in the bioaugmented reactors, it markedly increased to 38.00%, 68.20%, and 74.84% in R1, R2, and R3, respectively, which was likely associated with the bioaugmentation of strain FX1. The marginal increase between R2 and R3 further suggested that *Acinetobacter* enrichment was possibly constrained by substrate availability, ecological niche saturation, and microbial competition. Notably, despite the low initial abundance of *Acinetobacter*, substantial nitrogen transformation was still observed within 72 h in R0, indicating that the indigenous microbial community also played a critical role in nitrogen conversion. Functional inference from sequencing data revealed that the indigenous sludge harbored multiple nitrogen-cycling-related bacteria. Synergistic interactions between *Candidatus Competibacter* and *Candidatus Accumulibacter* have been reported to contribute to coupled denitrification and biological phosphorus removal processes [53]. In addition, the presence of *Saprospiraceae* [54], *Pseudomonas* [54], *Terrimonas* [55], *Defluviicoccus* [56], and *OLB13* [57] has been suggested to be likely beneficial for nitrogen removal. The introduction of strain FX1 further enhanced this process by strengthening ammonium assimilation capacity, resulting in improved overall nitrogen removal efficiency. Collectively, these results suggest that nitrogen transformation in this system was governed by synergistic interactions between strain FX1 and the indigenous microbiota.

## 4. Conclusions

A novel cold-resistant *Acinetobacter pragensis* FX1 was successfully isolated and exhibited efficient nitrogen removal over a broad temperature range. Nitrogen balance and genomic analyses consistently demonstrated that inorganic nitrogen was predominantly retained through assimilation rather than gaseous conversion. The presence of cold shock proteins and fatty acid biosynthesis-related genes further provided a potential genetic basis for sustained metabolic activity under low-temperature conditions. Moreover, bioaugmentation with strain FX1 enhanced treatment performance through synergistic interactions with indigenous microorganisms in activated sludge. These findings highlight the feasibility of employing strain FX1 for low-temperature wastewater treatment and provide a sustainable strategy for simultaneous nitrogen removal and recovery.

## Figures and Tables

**Table 1:** The specific growth rate and generation time of strain FX1 under different temperatures.

Temperature (°C)	*k* (h^−1^)	*g* (h)
5	0.04	18.09
10	0.06	11.64
20	0.11	6.26
30	0.20	3.46

**Table 2:** Nitrogen balance in various media.

Medium	Initial Nitrogen (mg/L)	Final Nitrogen (mg/L)						
		Extracellular Nitrogen				Intracellular Nitrogen		Nitrogen Loss
		NH_4_^+^-N	NO_2_^−^-N	NO_3_^−^-N	Extracellular Organic-N	Intracellular Soluble TN	Biology Nitrogen	
HNM	48.52 ± 0.36	0.56 ± 0	0.03 ± 0.01	0.60 ± 0.07	3.49 ± 0.48	24.21 ± 1.29	19.33 ± 0.14	0.30 ± 0.02
ADM-1	53.13 ± 1.66	-	0.57 ± 0.02	10.45 ± 0.60	1.90 ± 1.43	25.29 ± 3.24	14.55 ± 2.06	0.37 ± 0.15
ADM-2	49.91 ± 1.60	0.56 ± 0.28	2.15 ± 0.03	-	1.30 ± 0.39	30.57 ± 2.66	15.31 ± 2.09	0.02 ± 0.01

Values represent the mean ± SD of triplicate experiments. - denotes undetectability.

**Table 3:** Genetic information for strain FX1.

Sample ID	Strain FX1
Gene No.	3884
Genome size (bp)	4,054,481
Gene total length (bp)	3,465,834
Gene average length (bp)	892.34
Gene density genes (kb)	0.96
G + C (%)	43.68
Gene/Genome (%)	85.48
rRNAs	21
tRNAs	86

## Data Availability

The original contributions presented in this study are included in the article/Supplementary Material. Further inquiries can be directed to the corresponding author. The 16S rDNA gene sequences of strain FX1 were submitted to the NCBI GenBank database under accession number PP976548. The whole-genome sequence of strain FX1 has been deposited in the NCBI database under BioProject accession number PRJNA1395770. All sequence data generated from activated sludge microbial communities (S0 and R0–R3) are available in the NCBI Sequence Read Archive (SRA) database under BioProject accession number PRJNA1482743.

## Supplementary Materials

The following supporting information can be downloaded at: https://www.mdpi.com/article/10.3390/microorganisms14071529/s1, Text S1. Source of activated sludge samples and pretreatment procedures; Text S2. The procedures for scanning electron microscopy; Text S3. 16S rDNA gene sequencing analysis of strain FX1; Text S4. Complete genome sequencing of strain FX1; Text S5. Microbial community analysis; Figure S1. The final NO_3_^−^-N concentration and growth of nine preliminarily selected strains after 72 h of cultivation; Figure S2. The circular genome maps of Plasmid A (A) and Plasmid B (B) from strain FX1; Table S1: Components of media; Table S2: Detailed table of key gene information of nitrogen metabolism in strain FX1; Table S3: Detailed table of cold tolerance gene in strain FX1. References [58,59,60,61,62] are cited in the supplementary materials.
