# Supplementary material for: Nitrogen Removal Characteristics and Application by a Novel Cold-Resistant Bacterium
Source: Microorganisms. 2026 Jul 13;14(7):1529. doi: 10.3390/microorganisms14071529 (PMC13414396; doi:10.3390/microorganisms14071529)
Supplement: Supplementary file 1 [file microorganisms-14-01529-s001.zip › microorganisms-4408452-supplementary-final.pdf]

Supplementary Materials

Text S1. Source of activated sludge samples and pretreatment procedures

These municipal wastewater treatment plants are located in northern Shaanxi, China. During winter, the wastewater temperature is typically maintained at 7.0–12℃. All plants primarily treat domestic sewage, and the effluent meets the Level A criteria in the *Integrated Wastewater Discharge Standard of Yellow River Basin in Shaanxi Province* (DB 61/224-2018). The treatment systems are mainly based on multistage A/O or A^2/O configurations. To ensure stable performance under low-temperature conditions, several operational strategies were implemented, such as supplemental carbon addition, thermal insulation, and extended hydraulic retention time (HRT).

For sample pretreatment, sludge samples were transported to the laboratory under refrigerated conditions and processed immediately. These samples were centrifuged at 4000 rpm for 10 min, and the supernatant was carefully discarded. The resulting sludge pellets were resuspended with sterile deionized water and then centrifuged under the same conditions. This washing procedure was repeated three times. After the final washing step, the sludge samples were used for microbial enrichment and isolation.

Text S2. The procedures for scanning electron microscopy

The morphological characteristics of strain FX1 were examined by scanning electron microscopy (SEM) according to a previously reported method [58]. Freshly harvested bacterial cells were washed three times with 0.1 mol/L phosphate-buffered saline (PBS) and fixed in 2.5% (v/v) glutaraldehyde at 4°C for 12 h. After fixation, the cells were collected by centrifugation (5000 rpm, 3 min), washed three times with PBS, and sequentially dehydrated in a graded ethanol series (30%, 50%, 70%, 80%, 90%, 95%, and 100%), followed by drying out using a critical point dryer. Finally, the dried samples were coated with gold and observed under SEM at 3.0 kV.

Text S3. 16S rDNA gene sequencing analysis of strain FX1

Genomic DNA was extracted from strain FX1 using a Bacterial Genomic DNA Extraction Kit (Vazyme, #DC103). The resulting DNA was used as a template for PCR amplification with universal bacterial primers 27F (5′-TACGGYTACCTTGTTACG ACTT-3′) and 1492R (5′-AGAGTTTGATCMTGGCT CAG-3′). The amplification protocol consisted of an initial denaturation at 98℃ for 30 s, followed by 30 cycles of denaturation at 98℃ for 10 s, annealing at 54℃ for 20 s, and extension at 72℃ for 50 s. A final extension was performed at 72℃ for 5 min to ensure complete amplification. The PCR products were purified and subjected to Sanger sequencing. Raw chromatograms were inspected, edited, and assembled using SnapGene 6.0.2.

Text S4. Complete genome sequencing of strain FX1

Genomic DNA was extracted using a bacterial DNA extraction kit (magnetic beads) (Majorbio, Shanghai, China). The purity and concentration of the extracted DNA were assessed using a NanoDrop 2000 spectrophotometer (Thermo Scientific, USA) and a Quantus Fluorometer (Promega, USA), respectively. DNA integrity was verified by agarose gel electrophoresis. High-quality DNA samples were used for subsequent library construction. For Illumina sequencing, DNA samples were sheared into 400–500 bp fragments using a Covaris M220 Focused Acoustic Shearer. Illumina sequencing libraries were prepared from the sheared fragments using the NEXTFLEX Rapid DNA-Seq Kit. The prepared libraries were used for paired-end Illumina sequencing (2 × 150 bp) on Illumina NovaSeq X PLUS. For PacBio sequencing, genomic DNA was fragmented at ~10kb. DNA fragments were then purified, end-repaired, and ligated with SMRT bell sequencing adapters (Pacific Biosciences, CA). Then, the PacBio library was prepared and sequenced on one SMRT cell. The data analyses were performed using the online platform of Majorbio Cloud Platform (http://cloud.majorbio.com). Raw data were processed with fastp v0.20.0 for quality control. The clean short reads were combined with HiFi reads and assembled into complete genomes using Unicycle v0.4.8 [59]. The coding sequences (CDs) of chromosomes and plasmids were predicted by Prodigal v2.6.3 [60] and GeneMarkS v4.3 [61], respectively. tRNA-scan-SE v2.0 [62] and Barrnap v0.9 (https://github.com/tseemann/barrnap) were applied to predict tRNA and rRNA, respectively.

Text S5. Microbial community analysis

Microbial genomic DNA was extracted using the E.Z.N.A.R Soil DNA Kit (Omega Bio-Tek, Norcross, GA, USA). DNA quality was verified by 1% agarose gel electrophoresis, and DNA purity was determined using a NanoDrop 2000 spectrophotometer (Thermo Scientific, USA). The bacterial 16S rRNA gene was amplified with primer pairs 338F (5'-ACTCCTACGGGAGGCAGCAG-3') and 806R (5'-GGACTACHVGGGTWTCTAAT-3'). PCR products were purified, quantified, pooled in equimolar amounts, and used for library construction before paired-end sequencing on the Illumina NextSeq 2000 platform (Illumina, San Diego, USA). Raw sequencing reads were quality-filtered and clustered into operational taxonomic units (OTUs) at 97% sequence similarity using UPARSE 7.1. Representative OTU sequences were taxonomically assigned against the Silva 16S rRNA database using the RDP Classifier v2.2 with a confidence threshold of 0.7. Subsequent bioinformatic analysis was performed on the online platform of Majorbio Cloud Platform (https://cloud.majorbio.com/).

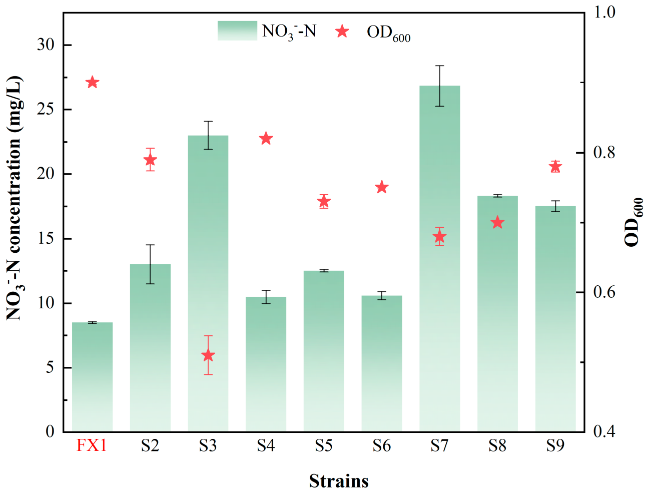

Figure S1. The final NO$_3^-$-N concentration and growth of nine preliminarily selected strains after 72 h of cultivation.

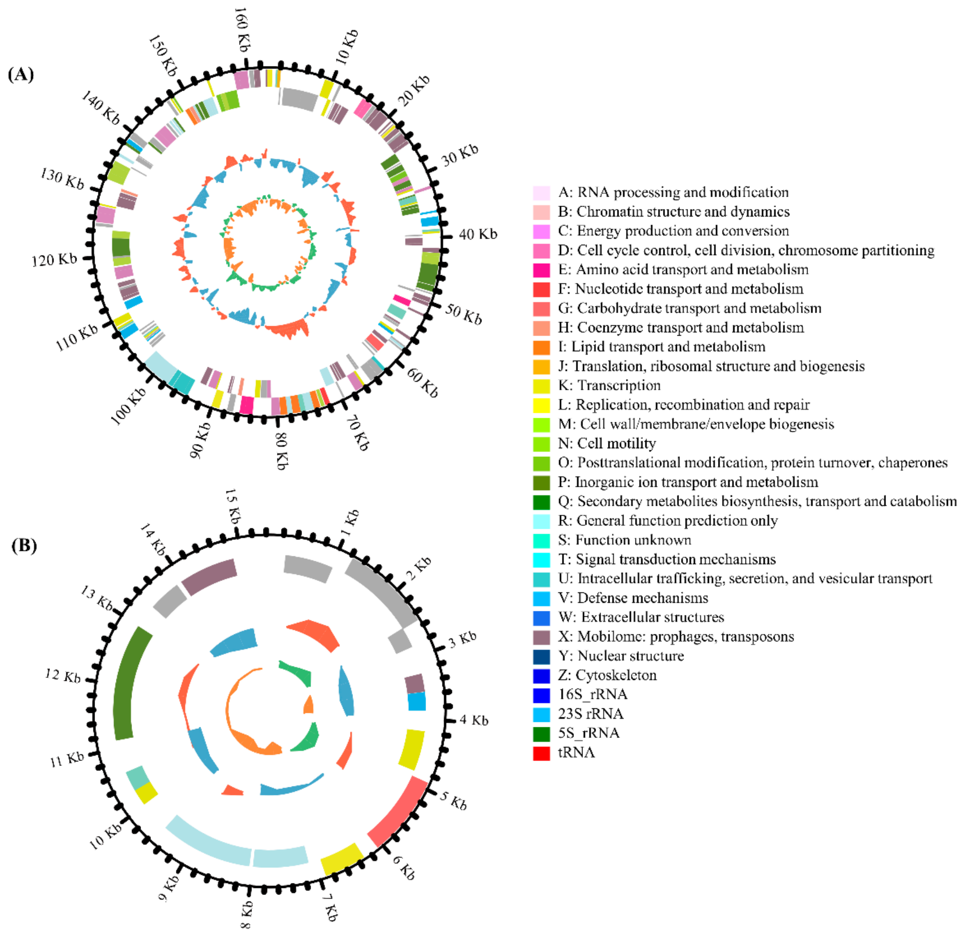

Figure S2. The circular genome maps of Plasmid A (**A**) and Plasmid B (**B**) from strain FX1. Annotations from the outermost to the innermost circles: the genome size scale (outermost circle), CDSs on the positive and negative strands (second and third circles), rRNA and tRNA (fourth circle), GC content (fifth circle), and GC-skew values (innermost circle).

Table S1. Components of media.

Medium	Composition and content of medium (g/L)										Trace element*
	peptone	yeast extract	NaCl	agar	sodium acetate	NH_4Cl	$NaNO_3$	$NaNO_2$	KH_2PO_4	$MgSO_4·7H_2O$	
LB	10	5	10	20							
BTB				20	1		0.303		0.045	0.1	1 mL
BM					1		0.303		0.045	0.1	1 mL
HNM					1.709	0.191			0.045	0.1	1 mL
ADM-1					1.709		0.303		0.045	0.1	1 mL
ADM-2					1.709			0.246	0.045	0.1	1 mL
SNDM-1					3.418	0.191	0.303		0.045	0.1	1 mL
SNDM-2					3.418	0.191		0.246	0.045	0.1	1 mL

*Trace element solution contained EDTA 5 g/L, $FeSO_4·7H_2O$ 5 g/L, H_3BO_3 0.05 g/L, $CuCl_2$ 0.03 g/L, $ZnCl_2$ 0.05 g/L, $(NH_4)_6Mo_7O_2·4H_2O$ 0.05 g/L, $CoCl_2·6H_2O$ 0.05 g/L, $AlCl_3$ 0.05 g/L, $NiCl_2$ 0.05 g/L, HCl 1 mL. All media were initially set to pH 7.0, and then autoclaved at 121℃ for 20 min.

Table S2. Detailed table of key gene information of nitrogen metabolism in strain FX1.

Gene ID	KO ID	KO Name	Definition
gene0251	K03320	amt	ammonium transporter, Amt family
gene0561	K03320	amt	ammonium transporter, Amt family
gene0967	K03320	amt	ammonium transporter, Amt family
gene0174	K15576	nrtA	nitrate/nitrite transport system substrate-binding protein
gene0352	K00265	gltB	glutamate synthase (NADPH) large chain [EC:1.4.1.13]
gene0353	K00266	gltD	glutamate synthase (NADPH) small chain [EC:1.4.1.13]
gene0968	K01674	cah	carbonic anhydrase [EC:4.2.1.1]
gene1973	K00372	nasC	assimilatory nitrate reductase catalytic subunit [EC:1.7.99.-]
gene1976	K01725	cynS	cyanate lyase [EC:4.2.1.104]
gene1977	K26139	nasD	nitrite reductase [NAD(P)H] large subunit [EC:1.7.1.4]
gene1978	K00362	nirB	nitrite reductase (NADH) large subunit [EC:1.7.1.15]
gene1979	K02575	narK	MFS transporter, NNP family, nitrate/nitrite transporter
gene2021	K01673	cynT	carbonic anhydrase [EC:4.2.1.1]
gene2905	K01673	cynT	carbonic anhydrase [EC:4.2.1.1]
gene2201	K00459	npd	nitronate monooxygenase [EC:1.13.12.16]
gene2594	K01915	glnA	glutamine synthetase [EC:6.3.1.2]
gene2764	K00262	gdhA	glutamate dehydrogenase (NADP+) [EC:1.4.1.4]

Table S3. Detailed table of cold tolerance gene in strain FX1.

Gene ID	KO ID	KO Name	Definition
			cold shock protein
gene2273	K03704	cspA	MULTISPECIES: cold-shock protein
gene3024	K03704	cspA	DNA-binding protein
gene3025	K03704	cspA	cold shock domain-containing protein
gene3567	K03704	cspA	DUF1294 domain-containing protein
gene3674	K03704	cspA	cold-shock protein
			Fatty acid biosynthesis
gene0240	K01897	fadD	long-chain acyl-CoA synthetase [EC:6.2.1.3]
gene0586	K00647	fabB	3-oxoacyl-[acyl-carrier-protein] synthase I [EC:2.3.1.41]
gene0587	K00647	fabB	3-oxoacyl-[acyl-carrier-protein] synthase I [EC:2.3.1.41]
gene0767	K00647	fabB	3-oxoacyl-[acyl-carrier-protein] synthase I [EC:2.3.1.41]
gene0588	K00059	fabG	3-oxoacyl-[acyl-carrier protein] reductase [EC:1.1.1.100]
gene0757	K00059	fabG	3-oxoacyl-[acyl-carrier protein] reductase [EC:1.1.1.100]
gene1378	K00059	fabG	3-oxoacyl-[acyl-carrier protein] reductase [EC:1.1.1.100]
gene1419	K00059	fabG	3-oxoacyl-[acyl-carrier protein] reductase [EC:1.1.1.100]
gene1541	K00059	fabG	3-oxoacyl-[acyl-carrier protein] reductase [EC:1.1.1.100]
gene1596	K00059	fabG	3-oxoacyl-[acyl-carrier protein] reductase [EC:1.1.1.100]
gene3374	K00059	fabG	3-oxoacyl-[acyl-carrier protein] reductase [EC:1.1.1.100]
gene3732	K00059	fabG	3-oxoacyl-[acyl-carrier protein] reductase [EC:1.1.1.100]
gene0589	K09458	fabF	3-oxoacyl-[acyl-carrier-protein] synthase II [EC:2.3.1.179]
gene0656	K01963	accD	acetyl-CoA carboxylase carboxyl transferase subunit beta [EC:6.4.1.2 2.1.3.15]
gene0756	K00645	fabD	[acyl-carrier-protein] S-malonyltransferase [EC:2.3.1.39]
gene2014	K02160	accB	acetyl-CoA carboxylase biotin carboxyl carrier protein
gene2015	K01961	accC	acetyl-CoA carboxylase, biotin carboxylase subunit [EC:6.4.1.2 6.3.4.14]
gene2135	K02372	fabZ	3-hydroxyacyl-[acyl-carrier-protein] dehydratase [EC:4.2.1.59]
gene3206	K01962	accA	acetyl-CoA carboxylase carboxyl transferase subunit alpha [EC:6.4.1.2 2.1.3.15]
gene3274	K00208	fabI	enoyl-[acyl-carrier protein] reductase I [EC:1.3.1.9 1.3.1.10]
gene2210	K22105	fabR	TetR/AcrR family transcriptional regulator, fatty acid biosynthesis regulator
gene3728	K22105	fabR	TetR/AcrR family transcriptional regulator, fatty acid biosynthesis regulator

References

58. Lu, Z.; Gan, L.; Lin, J.; Chen, Z. Aerobic Denitrification by *Paracoccus* Sp. YF1 in the Presence of Cu(II). *Sci. Total Environ.* **2019**, *658*, 81-86, doi:10.1016/j.scitotenv.2018.12.225.

59. Wick, R.R.; Judd, L.M.; Gorrie, C.L.; Holt, K.E. Unicycler: Resolving Bacterial Genome Assemblies from Short and Long Sequencing Reads. *PLoS Comput Biol* **2017**, *13*, e1005595, doi:10.1371/journal.pcbi.1005595.

60. Hyatt, D.; Chen, G.-L.; LoCascio, P.F.; Land, M.L.; Larimer, F.W.; Hauser, L.J. Prodigal: Prokaryotic Gene Recognition and Translation Initiation Site Identification. *BMC Bioinf.* **2010**, *11*, 119–129, doi:10.1186/1471-2105-11-119.

61. Besemer, J.; Borodovsky, M. GeneMark: Web Software for Gene Finding in Prokaryotes, Eukaryotes and Viruses. *Nucleic Acids Res.* **2005**, *33*, W451–W454, doi:10.1093/nar/gki487.

62. Chan, P.P.; Lowe, T.M. tRNAscan-SE: Searching for tRNA Genes in Genomic Sequences. In *Gene prediction*; Kollmar, M., Ed.; Methods in Molecular Biology; Springer New York: New York, NY, 2019; Vol. 1962, pp. 1–14 ISBN 978-1-4939-9172-3.
